# Normative values for relative schoolbag weight in primary school children aged 6-14 from Czech Republic: A pilot study

**DOI:** 10.1371/journal.pone.0225741

**Published:** 2019-11-25

**Authors:** Mario Kasović, Lovro Štefan, Martin Zvonar

**Affiliations:** 1 Department of General and Applied Kinesiology, Faculty of Kinesiology, University of Zagreb, Zagreb, Croatia; 2 Faculty of Sport Studies, Masaryk University, Brno, Czech Republic; Hochschule Trier, GERMANY

## Abstract

Little evidence from observational studies has been provided regarding ‘optimal’ relative schoolbag load during primary education. Also, no study to date has provided reference-based standards for relative schoolbag weight. Therefore, the main purpose of the study was to establish normative values of relative schoolbag weight in a sample of children. In this cross-sectional study, we recruited 584 primary school students aged 6–14 (mean_age_±SD = 9.6±2.4 yrs, mean_height_±SD = 1.4±0.2 m, mean_weight_±SD = 37.5±13.3 kg, mean_body-mass index_±SD = 17.6±3.1 kg/m^2^, 44.4% girls) chosen from five schools in the city of Brno. Schoolbag weight and child’s body weight were objectively measured by using digital scale. Relative schoolbag weight was calculated by dividing schoolbag weight with child’s body weight and the result was expressed in percentage. Lambda, Mu and Sigma (LMS) method was used to create sex- and age-percentile curves. Boys carried slightly heavier schoolbag, compared with girls (mean difference 0.2 kg, p = 0.020). No significant differences between sexes in relative schoolbag weight were observed (p = 0.240). Median values (P50) for boys and girls were similar and the largest observed between ages 6–9 in boys (15–17%) and 6–8 in girls (16–18%). The percentage of children carrying relative schoolbag weight beyond 10% of their body weight was very high, especially between ages 6–10 in boys (85.1–100%) and 6–11 in girls (86.8–95.4%). This study provides first sex- and age- relative schoolbag weight normative values in primary school children. Future studies should use similar methods for generating comparable data.

## Introduction

Children who enter primary education face an extreme external load for the first time. The majority of such load represents a schoolbag weight, which should be adapted according to their needs [1]. However, carrying a schoolbag represents a daily routine for children [2]. Although previous evidence has reported that a relative schoolbag weight must not exceed 10% of child’s body weight [3–5], recent evidence from Czech Republic has shown that 69.3% and 19.7% of children carry a schoolbag with relative weight of >15% and >20% of their body weight [6]. Some studies have shown negative effects of excessive schoolbag weight on back pain [2,7], prolonged blood pressure, oxygen uptake and energy expenditure recovery [8] and changes in kinematic, kinetic and electromyography parameters [9], while others have shown no evidence that schoolbag characteristics, such as weight, design or carriage method lead to back pain in children and adolescents [10]. Nevertheless, inappropriate load may lead to musculoskeletal pain which persists later in life affecting quality of life and seeking medical attention [11].

Although previous studies have defined a relative schoolbag weight [3–5] with potential shortcomings like different populations and methods studied [12], no study has established sex- and age-specific normative values for the same. Since schoolchildren spend most of the time in sedentary behaviors during school hours and schoolbag is the primary load for transferring books and accessories [1], such normative standards should be of crucial value for monitoring and adjusting relative schoolbag weight according to child’s needs and appropriate growth. Also, such parameters may also serve for comparing generated data from other countries and implementing school policies and strategies to reduce the level of schoolbag weight.

Therefore, the main purpose of the present study was to establish normative values of relative schoolbag weight in a sample of 6–14 year-old children.

## Materials and methods

### Study participants

In this cross-sectional study, participants were 6–14 year-old primary school children (mean_age_±SD = 9.6±2.4 yrs, mean_height_±SD = 1.4±0.2 m, mean_weight_±SD = 37.5±13.3 kg, mean_body-mass index_±SD = 17.6±3.1 kg/m^2^, 44.4% girls). When the study was conducted, there were 130 primary schools with approximately 400 students attending each school, giving an estimated number of 52 000 primary school children currently in the city of Brno, Czech Republic. Our sample size was estimated to be 583 by using 95% confidence level and 4% margin error. At the first stage, we randomly selected five primary schools with approximately 2 000 children. Before we began, we had contacted principals from each school to give permission for conducting the study. Our first sample size was 809 children. At the second stage, we introduced children and their parents with measurement protocol, potential contribution of the research, and possible discomforts during the execution of the research. Of these, 584 parents had given a written informed consent and those children were entered the study. Children were asked to bring schoolbag with school supplies for that school day, so that data on schoolbag weight and burden it represents for children would be accurate. All children were carrying schoolbag over both shoulders. All procedures were in accordance with the Declaration of Helsinki and approved by the Committee of the Faculty of Sports Studies.

### Measures

Body height was measured to the nearest millimeter in bare or stocking feet with the adolescent standing upright against a stadiometer (Seca, Japan). Body weight was measured to the nearest 0.1 kilogram and the participant wore light clothes with no shoes (Seca, Japan). BMI (kg/m^2^) was calculated as weight (in kilograms) divided by the square height (in meters). Schoolbag weight (in kilograms) was also recorded on digital scale. Relative schoolbag weight (in percentages) was calculated by dividing schoolbag weight with child's body weight.

### Data analysis

Age (without decimal places) and sex were self-reported. Basic descriptive statistics are presented as mean and standard deviation (SD). Sex differences were calculated by using Student *t*-test for independent samples. Spearman’s coefficient (r) was used to calculate the correlation between relative schoolbag weight and age. Next, we created smoothed sex- and age-specific 5^th^, 10^th^, 25^th^, 50^th^, 75^th^ and 90^th^ percentiles for relative schoolbag weight with Lambda (L), Mu (M) and Sigma (S) method. In this analysis, the optimal power to obtain normality is summarized by a smooth (L) curve and trends in the mean (M) and coefficient of variation (S) are similarly smoothed [13]. Next, all three curves (L, M and S) are summarized based on the power of age-specific Box–Cox power transformations for normalizing the data. Children carrying ‘optimal’ relative (defined as ≤10% of body weight) vs. ‘overload’ (>10% of body weight) schoolbag weight are presented in percentages for each sex and age group. All analyses were performed in Statistical Packages for Social Sciences (SPSS Inc., Chicago, Illinois, USA) with statistical significance of p<0.05.

## Results

Basic descriptive statistics of the study participants are presented in Table 1. As expected, boys were taller, heavier and had higher body-mass index values. Interestingly, boys also carried somewhat heavier schoolbag, compared with girls (p = 0.020). No significant differences in relative schoolbag weight between sexes were observed. The relative schoolbag weight significantly decreased by age in both boys (r = -0.53, p<0.001) and girls (r = -0.46, p<0.001).

**Table 1:** Basic descriptive statistics of the study participants, Czech Republic.

Measure	Total sample (N = 584)	Boys (N = 325)	Girls (N = 259)	*p*-value*
	Mean±SD	Mean±SD	Mean±SD	
**Age (yrs)**	9.6±2.4	9.8±2.4	9.4±2.4	0.015
**Height (m)**	1.4±0.2	1.4±0.2	1.4±0.1	0.022
**Weight (kg)**	37.5±13.3	38.9±14.3	35.7±11.8	0.004
**Body-mass index (kg/m**^**2**^**)**	17.6±3.1	17.9±3.3	17.2±2.9	0.009
**Schoolbag weight (kg)**	4.9±1.3	5.0±1.3	4.8±1.3	0.020
**Relative schoolbag weight (%)**	14.3±4.7	14.1±4.5	14.5±4.9	0.240

*Student *t*-test for independent samples *p*<0.05

**Fig 1:**
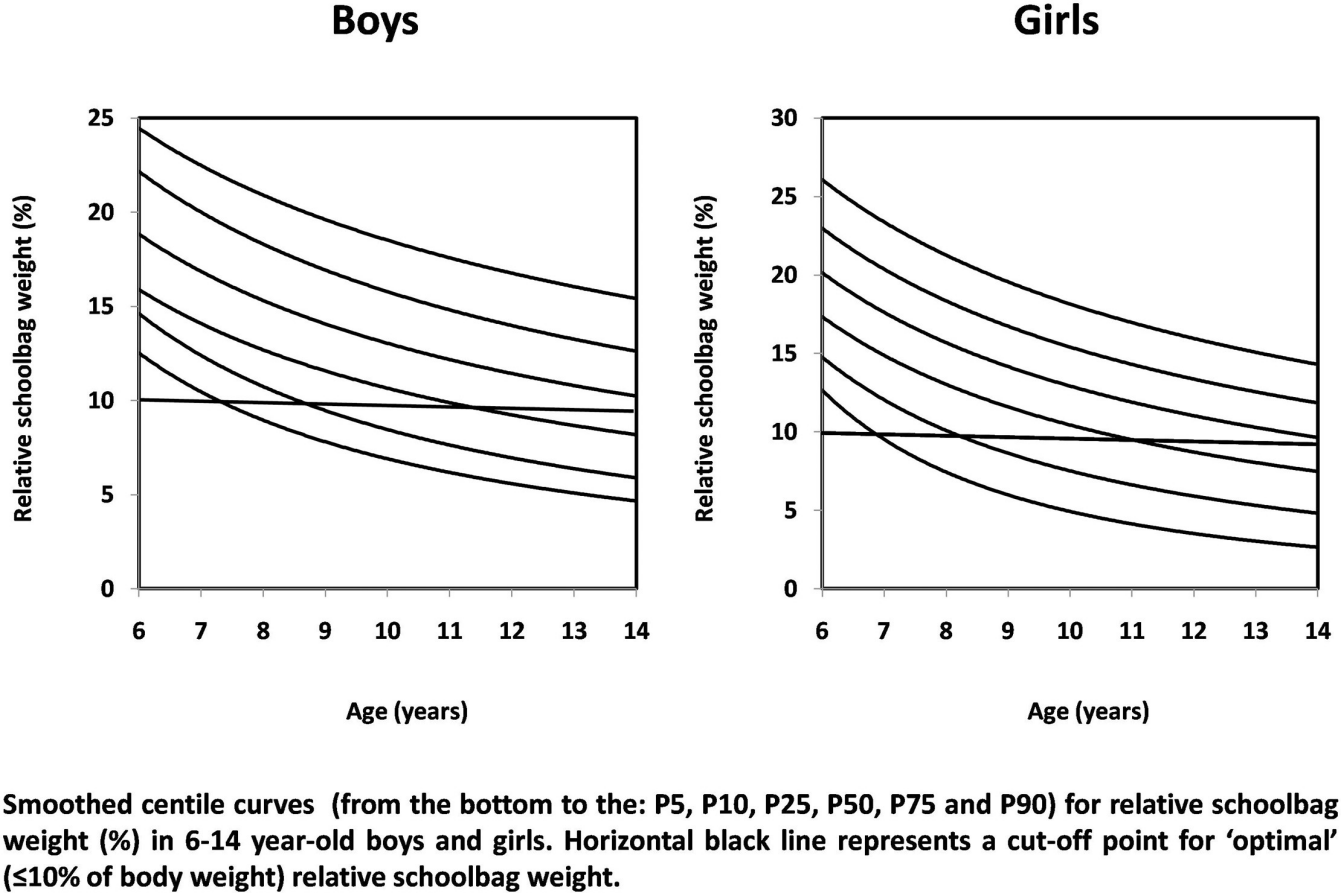
Sex- and age-specific normative values for relative schoolbag weight, Czech Republic.

Table 2 shows sex- and age-specific normative values for relative schoolbag weight. The same data are graphically presented in Fig 1. In boys, the median value (P50) ranged between 9–17%, with the highest relative schoolbag weight percentage during the first four grades of primary school, after which the percentage significantly decreased. In girls, the median value ranged between 8–18%, with the highest relative schoolbag weight percentage between ages 6–8 and the lowest values during final grades of primary school. The relative schoolbag weight in the highest percentile (P90) was between 14–23% in boys and 11–23% in girls.

**Table 2:** Normative values for relative schoolbag weight (%) of the study participants, Czech Republic.

Sex	Age	N	Percentile					
			P5	P10	P25	P50	P75	P90
**Boys**	6	18	11	13	15	17	21	23
	7	47	11	13	14	17	20	23
	8	44	7	9	12	15	18	21
	9	52	9	11	13	16	18	20
	10	35	10	11	12	14	17	19
	11	31	7	8	10	13	15	18
	12	47	6	7	9	12	15	19
	13	22	4	6	8	9	11	14
	14	29	4	5	8	10	13	15
**Girls**	6	29	10	11	14	18	20	23
	7	43	10	12	14	16	18	21
	8	38	6	10	15	17	19	21
	9	28	9	11	12	15	18	23
	10	37	6	10	13	15	19	20
	11	29	8	10	13	14	20	23
	12	23	2	5	8	12	15	19
	13	15	2	4	7	9	10	12
	14	17	3	4	6	8	9	11

The percentages of children carrying ‘optimal’ vs. ‘overload’ amount of relative schoolbag weight are presented in Table 3. In boys, the most prevalent ‘overload’ carriage was between the ages 6–10, after which the percentage dropped. Similar patterns were observed in girls, where the most prevalent ‘overload’ was between the ages 6–11. Moreover, a high percentage of older girls (13–14 year-olds) carried ‘optimal’ amount of schoolbag weight (86.7% and 94.1%), compared with boys (68.2% and 51.7%).

**Table 3:** Percentage of children carrying ‘optimal’ (≤10% of body weight) vs. ‘overload’ (>10% of body weight) amount of schoolbag weight, Czech Republic.

Sex	Age	N	Percentage of children	
			‘Optimal’	‘Overload’
**Boys**	6	18	0	100
	7	47	2.1	97.9
	8	44	14.9	85.1
	9	52	7.7	92.3
	10	35	5.7	94.3
	11	31	38.7	61.3
	12	47	29.8	70.2
	13	22	68.2	31.8
	14	29	51.7	48.3
**Girls**	6	29	6.9	93.1
	7	43	4.6	95.4
	8	38	13.2	86.8
	9	28	7.1	92.9
	10	37	10.8	89.2
	11	29	10.3	89.7
	12	23	47.8	52.2
	13	15	86.7	13.3
	14	17	94.1	5.9

## Discussion

The main purpose of the study was to establish age- and sex- normative values for relative schoolbag weight in a sample of 6–14 year-old children. Our study shows that the majority of children, especially in lower grades are at extreme risk of ‘overload’ schoolbag carriage. We also found that children, in the lowest percentile (P5), carried a relative schoolbag weight of >10% of their body weight.

Previous studies have reported that the relative schoolbag weight of >10% was prevalent in 4% to 61% of children [6,14,15]. Although recent evidence has shown that schoolbag characteristics; i.e. weight, design and carriage method are not significantly associated with back pain in children and adolescents [10], children who enter primary education with no past excessive loading may be at extreme risk of developing future diseases [11]. To date, no concrete policies and strategies have been taken with respect to it yet [16]. A schoolbag weight-reducing study by Sjazwan et al. [5], showed that children in the intervention group reported significantly less musculoskeletal problems and improved sitting posture, concluding that such ergonomic educational programs might be effective strategies for improving health.

Our results also show a significant inverse correlation between relative school bag weight and age for both boys and girls. However, according to Fig 1, children between ages 6–11 carried the largest amount of relative schoolbag weight. In boys, the first percentile curve crossed the horizontal black line at the age of 7 and continued to decrease till the age of 14. Beyond the first one, the second (P10) and the third (P25) percentile curve crossed the horizontal black line at the age of 8.5 and 11.5 and continued to decrease till the age of 14. The same patterns were observed in girls. Such results have been confirmed previously [6]. Specifically, Kasović et al. [6] showed that >90% of children in the first three grades of primary school carried a schoolbag >10% of their body weight. The same group of authors also found that the peak plantar pressure and contact surface for the forefoot, midfoot and hintfoot significantly increased when children were carrying a schoolbag, compared with no load [6]. Interestingly, the median percentile curve (P50) did not cross the horizontal black line across the age group, pointing out that primary school children beyond the 25^th^ percentile are at extreme risk of relative schoolbag ‘overload’. We also observed a significant decline in relative schoolbag ‘overload’ at the age of 13 and 14 in both sexes. In this specific time period, both growth and weight rapidly increase, reaching a peak at about 12 years in girls and 14 years in boys [17,18].

This study has a few limitations. First, by using a cross-sectional design, we cannot provide the maturation status and children’s growth. Second, we only included children from five primary schools in the city of Brno, limiting the generalizability of our results to other populations (mixed or rural) and ethnicity. Third, before the study began, we had introduced the parents about the main purposes of the study, which might have led to potential bias and higher drop-out rate. Therefore, future studies aiming to establish similar reference-based percentile curves for relative schoolbag weight should be longitudinal with larger sample sizes.

## Conclusions

Our study shows the first normative values for relative schoolbag weight in a relatively large sample of primary school children from Brno, Czech Republic. Findings from this study should be taken into account when establishing panel surveys and creating special strategies and policies in determining the ‘right’ amount of relative schoolbag weight and ergonomic features of the schoolbag load in a specifically risky group of 6–10 year-old children.

## Supporting information

S1 Raw Data (XLSX)
